# Exploring the effects of polymorphisms on *cis*-regulatory signal transduction response

**DOI:** 10.1016/j.molmed.2012.11.003

**Published:** 2013-02

**Authors:** Alasdair MacKenzie, Benjamin Hing, Scott Davidson

**Affiliations:** Gene Regulatory Systems Laboratory, School of Medical Sciences, Institute of Medical Sciences, University of Aberdeen, Aberdeen, Scotland AB25 2ZD, UK

**Keywords:** stratified medicine, gene regulation, *cis*-regulatory sequence, signal transduction, single nucleotide polymorphism, context dependency, personalized medicine, epigenetic modification

## Abstract

*cis*-Regulatory sequences (CRSs) direct cell-specific and inducible gene expression in response to signal transduction networks, and it is becoming apparent that many cases of disease susceptibility and drug response stratification are due to polymorphisms that alter CRS responses in a context-dependent manner. In the current review, we describe successful methods for identifying CRSs and analyzing the effects of allelic variation on their responses to signal transduction. The technologies described build on the successes of ENCODE (ENCyclopedia Of DNA Elements) by exploring the effects of polymorphisms on CRS context dependency. This understanding is essential to uncover the genomic basis of disease susceptibility and will play a major role in delivering on the promise of personalized medicine.

Meta-analyses of multiple genome wide association (GWA) (see Glossary) studies have shown that 88% of disease-associated single nucleotide polymorphisms (SNPs) are found within intronic or intergenic DNA [1,2], and GWA studies designed to detect the causes of drug response stratification [3] or unwanted side effects suggest a major role for intronic and intergenic variations [4–8]. Although many of these characteristics are associated with nonfunctional SNPs, many of these sequence differences occur within genomic regions of strong linkage disequilibrium (LD) that contain functional SNPs, the majority of which are noncoding [9]. Furthermore, a large proportion of GWA study hits are found within gene deserts that contain no genes, suggesting a role for long-distance gene regulation in disease. By describing the human regulatory landscape in unprecedented detail using many different cell culture studies, the ENCODE (ENCyclopedia Of DNA Elements) consortium have thrown down the gauntlet to biologists to determine the regulatory causes of diseases [10–13]. The next step in this process is to consider how polymorphic variation within the *cis*-regulatory genome alters its activity in response to signal transduction cues that represent the most important step in the transfer of biological information from the cell surface to the genome (Figure 1).

This review examines current techniques for identifying *cis*-regulatory sequences (CRSs) and describes methods that allow characterization of their context-specific activities. We review methods for establishing the effects of polymorphisms on the ability of CRSs to respond to signal transduction pathways in specific cell types, and then briefly summarize the possible influence of epigenetic modification on these processes. Finally, we examine several recent endeavors to investigate the effects on gene regulation of noncoding SNPs, identified by GWA studies of disease and drug nonresponse.

## Classifying CRSs

**Figure 1:**
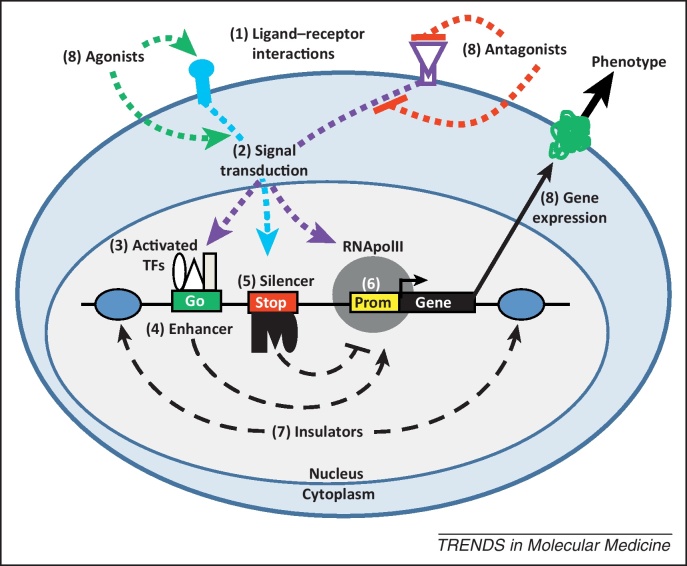
A highly simplified diagrammatic representation of a gene regulatory system demonstrating the general flow of information within eukaryotic cells and the points of interaction of cell signaling agonists and antagonists.

The ENCODE consortium has very recently presented evidence that 4.5 times more regulatory information is present in the genome than information used to encode proteins [10]. This observation shows that the amount of functional biological information in the noncoding human genome exceeds that of the coding genome, which is entirely consistent with GWA analyses indicating that the noncoding genome is an important reservoir of disease causing or stratifying loci [1,2]. Many studies have suggested that more than half of the most conserved regions of the human genome consist of CRSs, which include promoters, insulators, enhancers, and silencers that are essential for the tissue-specific, temporal, and inducible expression of genes (Figure 1) [14,15].

Promoter regions are orientation dependent with respect to the genes they control and consist of a ‘core’ sequence bound by RNA polymerase II (RNApolII; Figure 1) and secondary sequences required to support some level of tissue-specific expression [16]. Two different promoter types have been characterized: large GC-rich, TATA-less promoters, generally associated with constitutively expressed genes, and smaller TATA box promoters that modulate the expression of genes with high levels of tissue-specific expression [10,16]. Using DNAse1 hypersensitivity mapping (Box 1), it has been shown that promoter regions contain a nearly invariant 50-bp region occupied by the core transcriptional apparatus (RNApolII and cofactors).

In contrast to promoters, enhancers and silencers are largely orientation and, to some extent, distance independent (Figure 1). Indeed, some enhancers, which upregulate gene expression, have been found to influence expression from a distance of 1 mega base (1 Mb) [17]. Recently, it has been discovered that between 62% and 74% of the genome is transcribed, including enhancers that generate transcripts called eRNA [11]. The role of eRNA is unclear, but it has been hypothesized that RNApolII, immobilized in nuclear transcription factories, pulls on the genome using transcription to manipulate enhancers and promoters into these factories [18,19]. By contrast, silencers characteristically downregulate gene expression by interacting with transcription factors (TFs) called repressors that, in turn, recruit corepressors to specifically modify histones and create a closed chromatin structure, obstructing transcription [20]. Finally, insulator sequences protect gene promoters from the regulatory influences controlling other genes [11].

Collectively, enhancers and silencers often work together to maintain the fine balance of tissue-specific promoter activity appropriate for health [21]. Indeed, the coordinated activity of many different enhancers and silencers may be required to facilitate appropriate promoter responses to the large number of information inputs received by the cell. These responses are achieved through a combination of forces: promoter binding by specific combinations of activated TFs or repressor proteins to regulate RNApolII activity through a process that involves DNA looping, often over many thousands of base pairs [22,23] (Figure 1). *In vivo* observations in transgenic mice indicate that many CRSs only function within very tight contextual limitations. For example, the TAC1 ECR1 element appears to only be active in a subset of cells in the amygdala, a region of the brain that controls fear behavior and may be involved in anxiety and depression [24], and the GAL5.1 enhancer could only support expression in specific groups of cells in both the amygdala and the hypothalamic paraventricular nucleus (PVN), which controls dietary choice and alcohol intake [25]. The proportion of cells in the human body in which these enhancers are active is less than 0.001% of all cells. Nevertheless, appropriate expression of the TAC1 and GAL genes in these tissues is essential for health, a fact supported by the extremely strong sequence conservation of ECR1 and GAL5.1 through evolution. Thus, it is likely that many CRSs are only active if given the correct combination of signals within the correct cell type, a phenomenon known as context dependency. It therefore follows that in many cases, the effects of polymorphisms on CRS activity will only be revealed using paradigms that reflect this context dependency. Understanding how regulatory polymorphisms influence context dependency will be the next major step in understanding the basis of disease and drug response stratification.

## CRS and signal transduction relationships

Tissue-specific gene expression relies on the context-dependent activity of CRSs. In turn, context dependency relies on the relaying of contextual information, in the form of specific types of cell–cell communication at the cell surface (e.g., ligand–receptor interactions), to RNApolII at gene promoters. Activation of cell surface receptors by ligand binding subsequently activates signal transduction cascades. Many hundreds of different ligand–receptor interactions have been identified, and their effects on the many different known signal transduction networks have been widely published, which include the following canonical examples: kinase A and C pathways [26,27], the different mitogen activated protein kinase (MAPK) pathways (MEKK/ERK, p38 and JNK) [28], the JAK/STAT pathways [29], the Wnt pathway [30], and the tyrosine kinases [31], to name but a few. In addition to cell surface proteins, nuclear receptors (NRs) act as both receptors and TFs and form another layer of influence on gene regulation. Examples of NRs include the sex hormone receptor, vitamin D receptors, and stress hormone receptors (glucocorticoid and mineralocorticoid receptors) [32].

The chain of events leading to the activation and/or repression of RNApolII continues with the post-translational modification of TF proteins by activated signal transduction pathways, leading to the phosphorylation, glycosylation, or proteolytic digestion of TF proteins. This is a critical step in gene regulation, because a significant majority of TFs need to be activated by signal transduction pathways before they can bind CRSs, modify chromatin, or influence RNApolII activity (Figure 1) [33]. Indeed, it is now accepted that a major influence on disease susceptibility and drug response stratification centers on polymorphisms within CRSs that alter interactions with TFs. A vast literature describes the biochemistry and interactions of signal transduction networks, and the protein–DNA interactions that modulate gene expression. However, almost nothing is known about the effects of CRS polymorphisms on their ability to respond to signal transduction cues. In the wake of the significant advances made by the ENCODE consortium, filling this major knowledge gap is the next critical step in understanding the mechanisms through which CRS polymorphisms affect disease susceptibility and drug response stratification.

## Identifying a CRS

**Figure 2:**
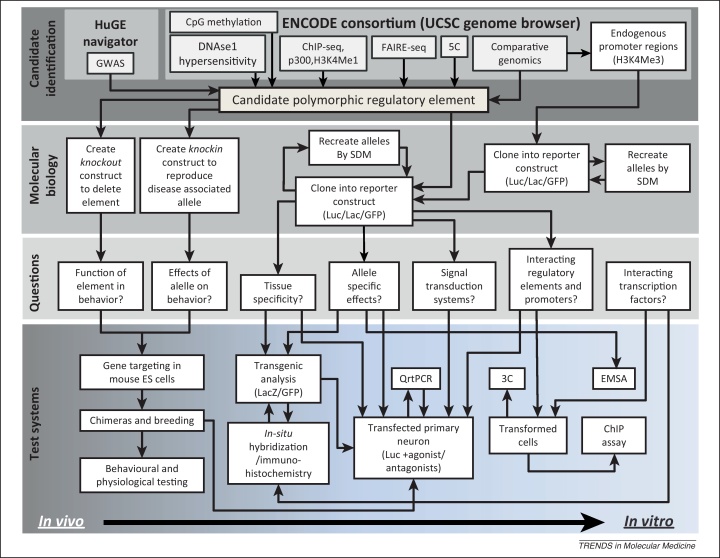
A flow diagram describing the relationships between different technologies that can be used to identify and characterize *cis*-regulatory sequences (CRSs) and to determine the effects of polymorphisms on their qualitative and quantitative activities using a series of different *in vivo*, *in vitro*, and high-throughput technologies. The first row describes the technologies that can be used to identify CRSs (GWAS, genome-wide association analysis; ChIP-seq, chromatin immunoprecipitation sequencing; FAIRE-seq, formaldehyde-assisted identification of regulatory element sequencing; 5C, carbon copy chromatin conformation capture). The second row describes the genome and DNA manipulations required to test hypotheses relating to CRS activity and the effects of polymorphisms (SDM, site-directed mutation; Luc, luciferase; Lac, *LacZ* gene encoding β-galactosidase; GFP, green fluorescent protein). The third row summarizes many of the different questions relevant to the understanding of the function of CRSs and the effects of SNPs on their activity. The last row summarizes several different paradigms that can be used to address the questions posed in the third row (ES, embryonic stem cell; QrtPCR, quantitative reverse transcriptase polymerase chain reaction; 3C, chromatin conformation capture; EMSA, electrophoretic mobility shift assay; ChIP, chromatin immunoprecipitation). This flow diagram is not exhaustive and does not include technologies that allow analysis of epigenetic modification.

The main obstacle to understanding the mechanisms that affect CRS activity has been our inability to identify them. In the past, identifying CRSs has relied on the painstaking deletion analysis of gene flanking regions. However, thanks to the sequencing of the human genome and the availability of ever more rapid advances in high-throughput sequencing technologies, several more rapid and effective high-throughput solutions have been devised to find and characterize CRSs (summarized in Box 1 and Figure 2).

These methods include formaldehyde-assisted identification of regulatory elements and next generation sequencing (FAIRE-seq) and DNAseI-seq analysis, which identify transcriptionally active parts of the genome that are less tightly associated with histone proteins and so are more susceptible to mechanical and enzymatic fragmentation, respectively [13,34]. A total of 2.9 million regions of the human genome have been identified as DNAse1 hypersensitive sites (DHS), vastly outnumbering known genes (∼23 000), and these are indicative of active *cis*-regulatory regions [13]. At higher resolution, DNAse1 digestion within DHSs can indicate the sites occupied by individual TFs – a technique known as genomic DNAse1 footprinting [12]. Methods such as 5C and Hi-C can identify interactions between different *cis*-regulatory elements at vast distances across the genome [22,35]. ChIP-seq is another technique that has been used by ENCODE to identify interactions between modified histones and TFs throughout the genome [36]. The use of ChIP-seq has identified a complex ‘chromatin signature’ based on the occurrence of different methylation and acetylation states for specific amino acids in different histones such as histone 3 (H3). For example, it has been shown that monomethylation of lysine 4 (K4) is a characteristic of H3 proteins that associate with enhancer regions (H3K4Me1). Likewise, H3 with trimethylated K4 is associated with promoters (H3K4Me3), and trimethylation of either H3 Lysine 27 or lysine 9 (H3K27Me3 and H3K9Me3, respectively) is associated with silenced chromatin [37]. Other ChIP-seq experiments have suggested that the binding of cofactors such as p300 and mediator are indicative of enhancers [38].

The techniques described above represent levels of investment in technologies and expertise that only a few specialized laboratories possess. An alternative to the experimental identification of *cis*-regulatory elements is the use of *in silico* prediction based on information currently available in online databases. One such *in silico* technique is comparative genomics (Figure 2), which relies on the hypothesis that sources of information in the genome important for survival are conserved throughout evolution [39]. Using comparative genomics, it has been observed that sequences that are deeply conserved – where depth of conservation takes into account both evolutionary time and sequence homology – such as that observed between humans and fish (400 million years) often represent early embryonic enhancer sequences [14,15,40]. However, these embryonic enhancers are significantly less polymorphic, thus reducing their usefulness in detecting the heritable causes of human disease [41]. Instead, a more fruitful search for disease-causing regulatory polymorphisms may lie in examining the conservation between less divergent genomes such as birds and humans [25,42,43] or mice and humans [21]. Initial findings from the ENCODE consortium suggest that only 40% of conserved noncoding sequences are functional [44], and these conclusions have somewhat reduced the perceived usefulness of comparative genomics in the identification of CRSs. However, based on the evidence of strong context dependency shown by many CRSs, it cannot be ruled out that the functionality of much of the remaining 60% of the conserved genome remains undetected because of the limitations of the cell culture-based paradigms used by the early ENCODE project.

## Paradigms for analyzing CRS function and signal transduction response

The staggering amount of data generated by the ENCODE consortium has been made publicly available through the University of California, Santa Cruz (UCSC) genome browser (http://genome.ucsc.edu/index.html), ENSEMBL (http://www.ensembl.org/index.html), and the ENCode project website (http://encodeproject.org/ENCODE/). These represent one of the greatest platforms ever made available to the scientific community [10]. However, the publication of this resource represents only the very beginning of our understanding of the regulatory genome. The majority of the conditions that affect the aging human population stem from processes that arise later in life and have high degrees of cell specificity. Because the vast majority of the CRSs identified by the consortium have largely been described in homogenous cell culture, a necessity for the genome-wide high-throughput techniques used, it remains to be determined what proportion of the CRSs identified by ENCODE possess the levels of context dependency required for appropriate cell-specific gene expression *in vivo*.

The ‘gold standard’ for determining the function of predicted CRSs, or the effects of polymorphisms on their activity, is analyzing the effect of their physical deletion from the genome or reproducing specific disease-associated alleles in living organisms. This is currently carried out using embryonic stem (ES) cell targeting to produce what are often referred to as ‘knockout’ mice. Mouse models are living-four dimensional test beds that share many physiological characteristics with humans. However, the use of ES cell targeting in mice is time consuming, technically challenging, and expensive (Figure 2) [45]. Therefore, before considering the production of CRS knockout models or the reproduction of disease-associated alleles, exhaustive *in vivo* (through pronuclear microinjection of DNA into mouse 1 cell embryos as described later), cell-based, and *in vitro* studies must be undertaken to identify and characterize the most likely disease-causing polymorphic CRSs and their degree of context dependency (Figure 2).

### Promoter choice

CRSs are often unable to support the appropriate cell or inducible gene expression in the absence of a promoter region. Previously, CRS analyses have relied on the use of ‘generic’ or commercially available promoter sequences that have understandable advantages in terms of convenience. Although many groups have used generic promoters very successfully, observations of the requirement for enhancer–promoter combinations have to be addressed in the future, and this is best done using endogenous promoters [42,46–48]. It is also important to note that the ability of CRSs to upregulate or downregulate a given promoter is often context dependent and varies depending on the cell types used to assay their activity, the stimuli used, or the proximity of other regulatory sequences [49,50]. The relationship between the ECR2 enhancer and the TAC1 promoter is a good example; the ability of the TAC1 promoter to drive sensory neuron specific expression, or to respond to MAP kinase pathways or noxious stimuli, depends on the remote enhancer ECR2, which is itself inactive in the absence of the TAC1 promoter [42,51].

The alternative to using a generic ‘off the peg’ promoter is to use endogenous promoters of genes of interest (Figure 2). Following the same logic used to select remote CRSs, comparative genomics, the extent of CpG islands, and DNAse hypersensitivity mapping – information currently available through the ENCode consortium – can determine the extent of the endogenous promoter to be used [21,42,51]. Although cloning endogenous promoter regions to form bespoke reporter constructs is time-consuming, there are several clear and important advantages to their use:

1. Because an endogenous promoter maintains the expression of its gene, it is easier to reconcile the relevance of CRS effects on endogenous promoter activity to the role of that CRS in controlling the expression of the endogenous gene.
2. Use of endogenous promoters addresses the question of enhancer–promoter specificity [42,46,48].
3. Use of an endogenous promoter allows for the analysis of negative *cis*-regulatory regions whose importance in gene regulation and health is emerging [21].

#### Qualitative analysis

The expression of many genes crucial for health is often limited to a small number of specific cell types *in vivo*. Therefore, candidate CRSs must be analyzed to determine whether they are active within the same cell types as the genes they are presumed to control. The most cost-effective and rapid qualitative method of establishing the tissue-specific activity of CRS sequences is by producing transgenic animals via pronuclear microinjection of reporter gene constructs (Figure 2) [52]. The relevance of a candidate CRS to the expression of the genes they regulate is determined by colocalizing the expression of the reporter gene products, such as GFP or LacZ, with the mRNA or protein expressed from candidate genes (Figure 2). In many cases, much of the cell-specific activity of isolated human enhancer sequences analyzed using transgenic mice have accurately reflected the expression of the homologous endogenous gene in mouse. For example, analysis of the activity of the GAL5.1 enhancer, which lies 42 kb from the galanin gene, using fluorescent immunohistochemistry and *in situ* hybridization showed that expression of a LacZ reporter linked to the GAL5.1 enhancer was active in cells of the hypothalamic periventricular nucleus that also expressed galanin mRNA and protein [25].

Because of the random nature of transgene insertion, transgenic analysis cannot easily be used to quantify differences in the activity of polymorphic CRSs or the effects of allelic variants on CRS response to signal transduction cues [53]. A much more accessible and reproducible method involves the quantitative analysis in relevant tissue or cell cultures using reporter genes fused to generic or bespoke gene promoters (Figure 2).

The types of cells previously used for reporter assays include transformed cell lines such as COS and HeLa cells, which can be readily grown in the laboratory and easily transfected using standard protocols such as lipofection (Figure 2) [54]. However, these immortalized cells have undergone levels of phenotypic and genomic divergence from their progenitor cells that present problems when studying highly context-dependent CRSs. Primary cells represent an alternative and arguably more representative paradigm of the endogenous cell types found in the body and nervous system (Figure 2) [55]. Previous problems associated with the refractory nature of primary neurons to DNA transfection have been largely overcome using systems such as Amaxa transfection [56] or magnetofection [57]. In keeping with previous observations [58], we have found that the functional differences resulting from CRS polymorphisms are often cell type dependent and best assayed in several different primary cell types whose identity reflects the known expression profile of the endogenous gene (Figure 2) [21,42,43,51].

## Analyzing the effects of *cis*-regulatory variation on signal transduction response

It has not been widely considered that a major source of disease susceptibility and differences in drug efficacy may result from polymorphism-induced changes in the ability of CRSs to respond to signal transduction cues. Studies of the effects of different gene polymorphisms on gene expression in different regions of the brain have shown that the effects of these SNPs vary significantly between regions [58]. These studies are supported by our own observations that the effects of many SNPs within CRSs may not become obvious until appropriate signal transduction cues are present within the correct cell type [21]. Thus, given the right context, the effects of allelic variation in CRSs can often be significant in one cell type but not another. For example, the major polymorphic variant of a highly conserved CRS within intron 2 of the *CNR1* (cannabinoid receptor 1 gene) gene, which encodes the cannabinoid receptor, was inactive in primary hippocampal neurons. However, the minor variant, which was in strong LD with another SNP associated with obesity and addictive behavior, was highly active in hippocampal neurons and, in contrast to the major variant, responded robustly to the activation of MAP kinase pathways [43].

Exquisite balancing of tissue-specific gene expression and response to very specific stimuli seems to be a property of many genes that are essential to health. For example, overexpression or underexpression of the brain-derived neurotrophic factor (BDNF) gene produced similar depressive symptoms in knockout or overexpressing mouse lines and suggests that positive regulation of BDNF must be balanced by negative regulatory influences [59,60]. One of the best-studied BDNF promoters, BDNF prom IV, is highly active in the amygdala, hippocampus, and cortex, where it responds to stimuli such as cell depolarization and the activation of PKC and Wnt signaling pathways. However, a polymorphic silencer element, BE5.2, containing an SNP associated with depression (rs12273363) prevents activation of BDNF prom IV by PKC and Wnt-signaling pathways and only permits activation following cell depolarization or if both the PKC and PKA pathways are simultaneously stimulated [21]. In this respect, BE5.2 acts in a manner analogous to an ‘AND’ gate used in Boolean algebra and computational design in its ability to ‘filter’ the information contained in signal transduction cues. Significantly, the mood disorder associated C-allele of rs12273363 [61] increased the ability of BE5.2 to repress BDNF activation. Consistent with previous observations of region-specific effects of SNPs on gene expression in the brain [58], it has been observed that the C and T alleles of the BE5.2 silencer also behave differently in neurons derived from the amygdala: in contrast to hypothalamic and cortical neurons, the minor C-allele decreased the negative regulatory effects of BE5.2 on BDNF promoter IV [21].

## Gene regulation, CRSs and epigenetics

In recent years, it has become evident that changes in chromatin structure, which do not alter the primary sequence of the genome, induce phenotypic effects by altering gene regulation. These processes have been referred to as epigenetic modification and include chemical changes in the methylation status of DNA at CpG sequences or methylation and acetylation of histone proteins that can be inherited through many cell divisions and, purportedly, between human generations [62]. Although the precise mechanisms governing the heritability of these changes are yet to be established, it is highly likely that epigenetic modifications alter gene expression by changing the mode of action of associated CRSs. For example, early life stress resulted in hypomethylation of an enhancer that regulated arginine vasopressin (AVP) expression in mice [63,64], and hypomethylation of this AVP enhancer increased its activity, thus increasing expression of AVP in the PVN, an expression profile associated with depression [63,64]. Intriguingly, functional polymorphisms within the GAL5.1 and the CNR1 enhancer regions [25,43], which are candidates for controlling the expression of the mood, appetite, and pain modulating genes Galanin and cannabinoid receptor 1, have introduced or removed novel CpG sequences, respectively. Further examination of these loci using genome-wide methylation analysis through the ENCODE consortium web repository (http://genome.ucsc.edu/index.html) showed that the CpGs involved were methylated in these enhancers in cortical neurons, raising the possibility that functional genetic variance may interact with epigenetic modification to determine disease susceptibility (unpublished data). These observations are entirely consistent with previous studies of allele-specific methylation [65].

## Incorporation of GWA study data

In isolation, each of the techniques described above are unlikely to identify the causes of disease or stratification. It is essential that a multidisciplinary approach be used to further identify the regulatory causes of heritable diseases and stratification as described in Figure 2. The first steps in this process must be informed from GWA studies (Figure 2), many of which have been compiled in databases such as the HuGE navigator (http://hugenavigator.net/) or the NIH GWAS catalog (http://www.genome.gov/gwastudies/). For example, a few studies of critical diseases such as bipolar disorder (BD) and type 2 diabetes (T2D) have returned several GWA study hits that closely cluster and have shown strong reproducibility in many different GWA studies. All SNPs associated with BD and T2D are noncoding and occur in intronic or intergenic sequence. In the case of BD, multiple studies have identified noncoding SNPs within the *CACNA1c* and *ANK3* genes that demonstrate strong associations with BD [66]. Tantalizingly, many of these SNPs cluster within the third intron of the *CACNA1c* gene and occur within a region in strong LD that also includes several SNPs in highly conserved sequences. The risk allele of one of these SNPs (rs1006737) increased CACNA1C mRNA levels in patient lymphoblastoma cells [67] and brain [68], suggesting a CRS effect. However, at the time of writing, the authors were unaware of studies to determine the mechanisms through which the rs1006737 risk allele affects susceptibility.

Other strong GWAS disease associations include that between T2D and an SNP (rs7903146) within a repetitive region within intron III of the human *TCF7L2* gene [69]. Extensive comparative genomics and analysis of 92 kb of TCF7L2 intron III in transgenic mouse embryos using an exogenous heat shock promoter (Hsp68), succeeded in identifying multiple conserved regions that supported tissue-specific expression in the bone primordia, nervous system, and gut in embryonic day 15.5 mouse embryos [70]. However, determining the effects of the risk allele of rs7903146 on *cis*-regulatory activity is still to be achieved [70]. It is possible that examining SNPs in LD with rs7903146 within conserved regions using the endogenous TCF7L2 promoter in primary cells may prove revealing. Intriguingly, the products of TCF7L2, the Wnt-pathway activated transcription factor TCF4, demonstrate allele-specific binding to a cancer-associated SNP (rs698327) within a remote CRS that contacts and upregulates the activity of the c-MYC oncogene locus, which is located 335 kb away [71]. These elegant studies beautifully illustrate the efforts being made to reconcile noncoding polymorphisms with disease.

In the case of both the *CACNA1c* and *TCF7L2* genes, it is clear that we have reached a critical stage where the technologies described in the current manuscript, when combined with the ENCODE consortium databases, will make a real contribution to understanding the roles of BD and T2D risk alleles in altering expression of these genes. These examples demonstrate that we are currently on the threshold of a major advance in our understanding of the regulatory mechanisms governing the susceptibility and progression of many diseases with a major heritable component.

## CRS polymorphisms and drug response stratification

The development and testing of novel pharmaceuticals is all too often hampered by genetic heterogeneity, or stratification, in Phase II and III human test cohorts that greatly reduces the significance of test data and delays the delivery of novel compounds to the market. In addition, variations in the efficacy of, and side effects produced by, currently available drug therapies significantly impacts on the ability of health services around the world to deliver more effective personalized medicines. In order to expedite future market delivery of drugs in an efficient and timely manner, and to ensure the targeting of existing drugs to patients who would most benefit, the genetic determinants that cause drug response stratification must be identified. In addition to determining the causes of disease, GWA studies have also been used to find the causes of drug response stratification. Clues to the possible mechanisms through which polymorphisms in the noncoding genome affect drug response can be seen in the significant differences observed between the C and T allele of the CNR1 ECR1 enhancer; the T allele responds much more strongly to treatment using angiotensin II [43]. GWAS-based approaches to determining the parts of the genome responsible for response stratification include the Genome-based Therapeutic Drugs for Depression (GENDEP) study. The GENDEP consortium has used GWAS analysis to determine the causes of nonresponse to antidepressants and has identified several loci close to or within the uronyl 2-sulfotransferase (*UST*) and protein phosphatase 1A (*PP1*) genes that demonstrate strong association with nonresponse to the antidepressants escitalopram and nortriptyline, respectively [6,8]. Again, none of the SNPs associated with response occurred in coding regions and, in common with disease-causing SNPs, it is highly likely that the causes of escitalopram and nortriptyline nonresponse are regulatory. Thus, gaining a better understanding of the regulatory landscape surrounding the *UST* and *PP1* genes will likely be essential for understanding their role in drug response stratification.

## Concluding remarks

Thanks to the publication of the ENCODE consortium findings and the results of hundreds of GWA study data sets, there has never been a better time to understand how regulatory polymorphisms affect the expression of genes involved in disease susceptibility and drug response stratification. An opportunity now exists to build on the achievements of the ENCODE consortium and examine how disease-associated polymorphisms affect the activity of CRSs using paradigms that accurately reflect their context dependency. It will be interesting to determine how the ENCODE data, recovered from human ES cells, HeLa cells, or lymphoblastoma cells (human lymphocytes immortalized using viruses) translates to the tight tissue-specific expression of many genes in parts of the body such as the hypothalamus or the amygdala, that control appetite and mood, respectively, and where context dependency can be at its most extreme. It is hoped that by combining the ENCODE databases with the primary cell based and *in vivo* techniques described above, a level of understanding of the effects of SNPs or epigenetic modification in disease susceptibility and drug response will soon emerge. Only in this way will we be able to deliver on the promises of stratified and personalized medicine.

## Box 1 High-throughput technologies for the analysis of genome wide cis-regulatory interactions

Large-scale genomic analysis exemplified by the ENCODE consortium has been made possible by the development of next-generation technologies that sequence a major proportion of the DNA molecules in a given sample. In addition to producing sequence data, this technology can quantify the relative amounts of any given sequence within the DNA population. Techniques that use next-generation sequencing technologies are given a -seq suffix. For example, *RNA-seq* describes a technique for sequencing reverse-transcribed DNA generated from an mRNA sample, which allows not only the identification of the genes being expressed but also quantification of their relative amounts.

*FAIRE-seq*. ‘Formaldehyde-assisted isolation of regulatory elements’ exploits the phenomenon that, following formaldehyde treatment, transcriptionally ‘closed’ chromatin is relatively insoluble, whereas DNA in a more open configuration (i.e., less associated with histones) will remain more soluble and can be separated by phenol–chloroform extraction [72]. Subsequent high-throughput sequencing of DNA from the water-soluble fraction has been reported to provide an accurate portrait of transcriptionally active regions within the genome [72].

*DNAse1 sensitivity mapping-seq (DNAse-seq).* Although it does not involve formaldehyde treatment, DNAse-seq is similar in concept to FAIRE analysis in that it selectively breaks exposed, transcriptionally active DNA. Briefly, cells are lysed to release their nuclei that are then exposed to the DNAse1 enzyme, which is small enough to penetrate deep into the chromatin and digest any exposed DNA [73]. DNA from these nuclei is extracted and ligated to linker DNA that allows recovery and analysis by next-generation sequencing.

*5C* [74] *and Hi-C* [75]*.* These technologies detect interactions between widely separated sequences in the genome, such as remote *cis*-regulatory elements and promoters, using formaldehyde crosslinking. After formaldehyde crosslinking, chromatin is extracted from cells and exposed to restriction endonucleases. Extracted chromatin fragments are ligated at low concentrations and analyzed using next-generation sequencing. In this way, a ‘snapshot’ of chromatin folding in living cells and associations of widely separated parts of the genome such as promoters and long-range enhancers can be observed [76].

*ChIP-seq (chromatin immunoprecipitation-seq).* ChIP-seq analysis detects the binding of specific TFs to genomic DNA within living cells and starts with the formaldehyde treatment of cultured cells. Chromatin is then extracted from these cells and sheared into fragments by sonication. Enrichment of DNA fragments is carried out using antisera raised against specific DNA binding proteins, which also immunoprecipitates their bound DNA. The identity of this bound DNA is analyzed using next-generation sequencing that also determines relative quantities of immunoprecipitated DNA on a genomic level [77–79].

## Glossary

Amygdala: a region of the brain that controls fear-related behavior and mood. Misregulation of genes in this part of the brain may be involved in generating mood disorders such as chronic anxiety and major depressive disorder.
Brain-derived neurotrophic factor (BDNF): a secreted protein that acts on neurons of the central and peripheral nervous system; it supports the survival of existing neurons, and encourages the growth and differentiation of new neurons and synapses. Misregulation of BDNF expression has been linked to mood disorders and obesity.
Cannabinoid receptor 1 gene (*CNR1*): encodes the cannabinoid 1 receptor CB1. CB1 acts as the receptor for endogenous cannabinoids (anandemide) as well as plant-derived cannabinoids. CB1 plays a role in appetite, inflammatory pain, and modulating mood.
ChIP-seq: chromatin immunoprecipitation with next-generation sequencing (Box 1).
Chromatin conformation capture carbon copy (5C): a method of detecting long-range interaction between different sequences of the genome (Box 1).
*cis*-Regulatory genome: fraction of the human genome required to maintain levels of cell-specific and inducible gene expression appropriate for health.
*cis*-Regulatory sequence (CRS): noncoding sequences that control the expression of genes within specific cells, at specific amounts, and in response to specific stimuli. Include promoters, enhancers, silencers, and insulators.
Context dependent: *cis*-regulatory activity that is critically dependent on the correct cell phenotype, cell interaction, signal transduction cue, or genomic location.
DNAse1 hypersensitivity assay with next generation sequencing (DNAse-seq): a genomic technique for detecting enhancers and promoter sequences (Box 1).
ENCODE consortium (ENCyclopedia Of DNA Elements consortium): an international collaboration of research groups funded by the National Human Genome Research Institute (NHGRI).
Enhancer: a sequence of DNA that interacts with activated transcription factors and increases the activity of RNA polymerase II at a promoter region.
Formaldehyde-assisted identification of Regulatory elements and next generation sequencing (FAIRE-seq): a genomic technique for detecting enhancers and promoter sequences (Box 1).
Galanin (GAL): a secreted neuropeptide implicated in many biologically diverse functions, including inflammatory pain, cognition, feeding, and regulation of mood.
Genome wide association (GWA) study: commonly an examination of many thousands of common SNPs in thousands of different individuals to identify alleles associated with disease susceptibility.
Hypothalamus: a region of the brain that controls several physiological processes including appetite and thirst. Misregulation of genes in the hypothalamus may explain many cases of obesity.
Insulator: a region of DNA that insulates the promoter of one gene from the influences modulating the activity of a second gene promoter.
Intergenic: large areas of noncoding DNA between genes.
Linkage disequilibrium (LD): the nonrandom association of alleles at two or more loci. LD has proven to be very useful in GWA studies where an allele at one locus can be predicted by an allele at a second locus, thus removing the need to genotype all loci.
Preprotachykinin 1 (*TAC1*): a gene that encodes a secreted neuropeptide called substance P (SP). SP is expressed in dorsal root ganglia, where it controls inflammatory pain, and in the amygdala, where it plays a role in modulating mood
Post-translational modification: any modification of protein structure after translation. Includes phosphorylation, glycosylation, and proteolysis. In the case of transcription factors, post-translational modification is often mediated by signal transduction cascades resulting in their increased or decreased ability to bind DNA or modulate RNApolII activity.
Primary cell: an unmodified cell removed from a living animal and cultured alive under laboratory conditions.
Promoter: a sequence of DNA next to the transcriptional start site of a gene that acts as the initial binding site of RNApolII.
Signal transduction pathway: a cascade of specific protein modification events in the cytoplasm that is often initiated by activation of cell surface receptors and terminates in the post-translational modification of transcription factors in the nucleus.
Silencer: a sequence of DNA that reduces the activity of a promoter region.
Single nucleotide polymorphism (SNP): the most common polymorphisms in the genome generally affecting only one base pair to produce two or more alleles at a particular locus.
Stratified medicine: the analysis of the genetic causes of differences in drug response or disease susceptibility within populations.
Transfection: the process of introducing foreign DNA into single cells.
Transformed cell: a cell line that has undergone transformation to an immortalized ‘cancer-like’ state.
Transcription factors (TF): proteins that bind *cis*-regulatory sequences following their post-translational modification by signal transduction pathways. Once bound, TFs can influence RNApolII activity as well as modulating chromatin remodeling events.
